# Experimental and computational structure study of the commercial NMC622 lithium-ion battery electrode

**DOI:** 10.1557/s43578-026-01938-y

**Published:** 2026-08-05

**Authors:** Artur Braun, Alexey Rulev, Selma Erat, Murat Aycibin, Eda Uslu, Martin Winter, Sascha Nowak, Markys Cain, Iztok Arcon, Yves Ménesguen, Vladimir Pomjakushin, Paul Thompson, Burkhard Beckhoff

**Affiliations:** 1 Laboratory for High Performance Ceramics, Empa. Swiss Federal Laboratories for Materials Science and Technology, Überlandstrasse 129, 8600 Dübendorf, Switzerland; 2 Program of Opticianry, Department of Medical Services and Techniques, Vocational School of Technical Sciences, Mersin University, 33340 Mersin, Turkey; 3 Department of Nanotechnology and Advanced Materials, Institute of Science, Mersin University, 33340 Mersin, Turkey; 4 Physikalisch-Technische Bundesanstalt, 10587 Berlin, Germany; 5 University of Münster, Münster Electrochemical Energy Technology, MEET, 48149 Münster, Germany; 6 Electrosciences Ltd., Surrey, UK; 7 Laboratory for Quantum Optics, University of Nova Gorica, Vipavska 13, 5000 Nova Gorica, Slovenia; 8 Universite Paris-Saclay, CEA, LIST, Laboratoire National Henri Becquerel (LNE-LNHB), 91120 Palaiseau, France; 9 Laboratory for Neutron Scattering, Paul Scherrer Institut, Villigen PSI, 5232 Villigen, Switzerland; 10 University of Liverpool, Oxford St, Liverpool, UK

**Keywords:** Li, Energy storage, Oxide, Crystallographic structure, X-ray diffraction (XRD), Electronic structure

## Abstract

Reliable and traceable characterization of battery electrode materials is essential for standards and harmonized measurements in the growing lithium-ion battery sector. We report a coordinated interfacility study of commercial cathode NMC622, performed within the EURAMET projects OpMetBat and HyMetBat to evaluate reproducibility, uncertainty, and cross-method comparability. A single batch was analyzed using synchrotron X-ray diffraction, high-resolution neutron diffraction at two facilities, transition metal K-edge XANES/EXAFS, DFT-based electronic structure and spectral simulations, and electrochemical measurements. Neutron diffraction yields consistent lattice parameters and transition metal occupancies and enables traceable quantification of lithium content. Lithium loss from neutron refinement agrees with electrochemical charge extraction at low delithiation, whereas deviations at higher states of charge indicate parasitic faradaic processes. XANES and EXAFS confirm local coordination and oxidation states, while DFT reproduces spectral features. Operando impedance correlates with structural evolution and supports state-of-charge metrology. Together, these cross-facility results establish a robust metrological workflow for layered oxide cathode materials.

**Graphical abstract:**

**Figure:**
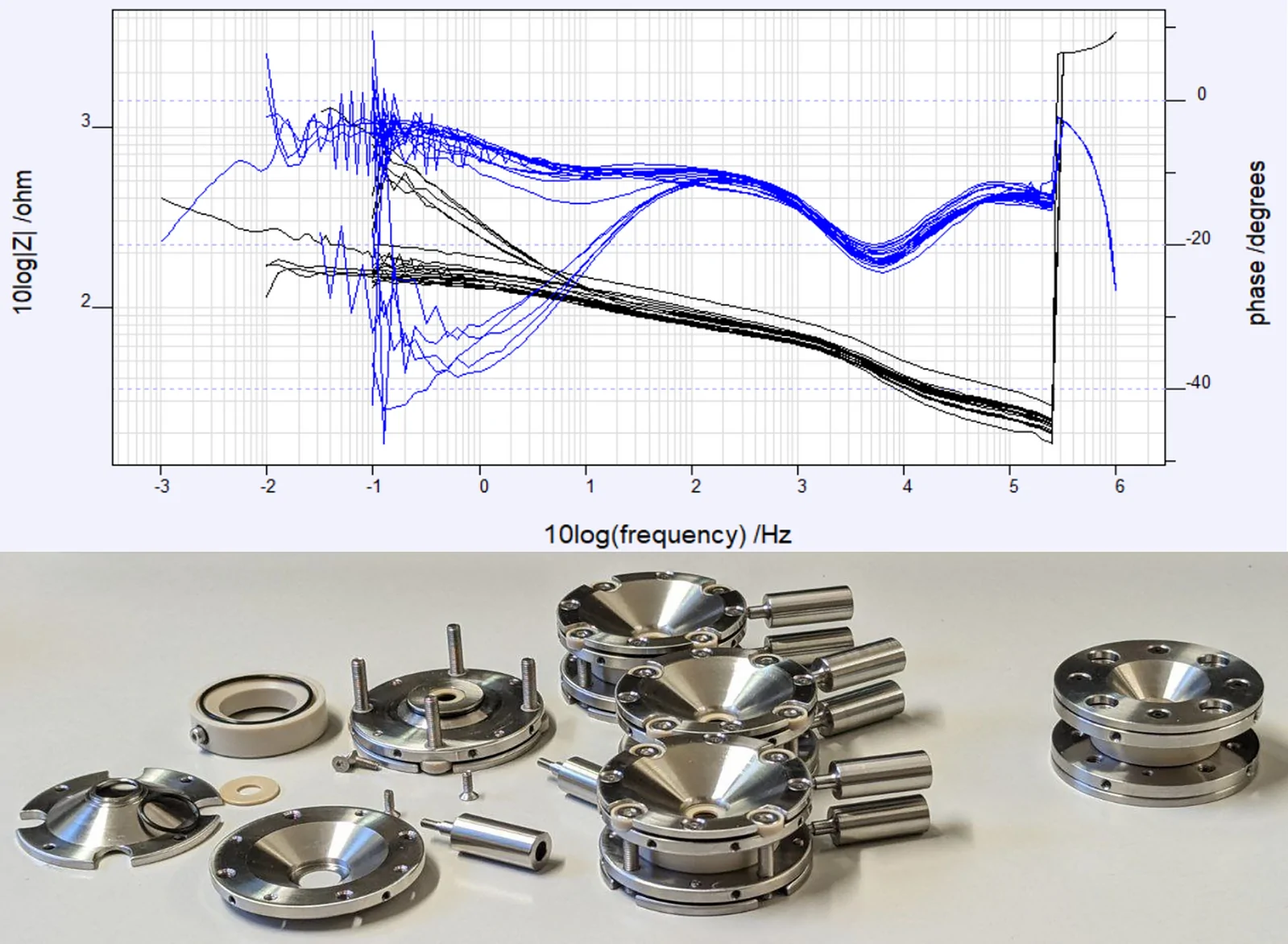

**Supplementary Information:**

The online version contains supplementary material available at https://doi.org/10.1557/s43578-026-01938-y.

## Introduction

The industrialization of lithium-ion batteries has created a strong demand for measurement standards, reference materials, and traceable characterization procedures capable of supporting manufacturing scale-up, safety regulation, and recycling workflows. In contrast to conventional structural materials, battery electrodes undergo continuous and state-dependent changes in composition, electronic structure, and lattice parameters during operation. Consequently, metrological descriptions must quantify not only static properties but also dynamic variables such as lithium content, transition metal oxidation state, oxygen stoichiometry, and lattice strain.

The concept of dynamic X-ray diffraction on operating lithium battery electrodes was pioneered by Chianelli et al. [1] in the late 1970s, who demonstrated real-time tracking of electrochemically induced structural changes during lithiation of TiS_2_. Synchrotron X-ray absorption and diffraction studies on working lithium-ion battery cathodes were first systematically demonstrated by McBreen and co-workers [2] in the mid-1990s. Building on these developments, our work introduced one of the first fully integrated electrochemical reaction cells enabling simultaneous X-ray scattering, diffraction, and spectroscopy on a functioning lithium-ion battery under realistic operating conditions [3–5]. These diagnostic approaches demonstrated that diffraction and spectroscopy can track electrochemically induced structural changes in real time, but they were not designed to address questions of traceability, interlaboratory reproducibility, or uncertainty propagation. The layered cathode material LiNi_0.6_Mn_0.2_Co_0.2_O_2_ (NMC622) [6] is widely used in both research and industrial applications and is commercially available as powder and processed electrode sheets [7–9]. Its established performance envelope reduced cobalt content, and relevance for electric vehicle applications made NMC622 an attractive candidate for developing transferable reference characterization workflows. Deviations in lithium stoichiometry, oxygen content, or cation mixing are known to induce significant changes in electrochemical performance and lifetime, with potential implications for intellectual property, quality control, and regulatory compliance [10, 11].

A broad body of literature has investigated NMC materials using experimental and theoretical approaches. Surface modification strategies, including protective and functional coatings, have been explored to improve cycling stability and high-voltage performance [12, 13]. Temperature-dependent structural and electrochemical behavior has been examined experimentally [14], while synchrotron-based spectroscopies have been employed to probe bulk and surface charge-compensation mechanisms [9, 15]. On the theoretical side, DFT has been widely used to investigate transition metal and oxygen redox behavior, electronic structure, elastic properties, and spectroscopic signatures in Ni-rich NMC cathodes [16–19], with comprehensive reviews summarizing computational progress in this field [6].

From a structural perspective, neutron diffraction (ND) offers high sensitivity to lithium and oxygen and enables quantitative determination of atomic site occupancies and lattice parameters. However, the reproducibility of refined structural parameters across different neutron instruments and facilities cannot be assumed a priori. Complementary techniques such as high-energy synchrotron X-ray diffraction (XRD), X-ray absorption spectroscopy (XANES/EXAFS), impedance spectroscopy, and density functional theory (DFT) calculations provide access to additional structural, electronic, and transport-related observables, yet their metrological comparability and mutual consistency require systematic assessment.

X-ray [20] and neutron diffraction are established as metrologically traceable techniques [21] when calibrated against certified reference materials (compare [22]) and when appropriate uncertainty budgets are defined. The same holds for X-ray absorption spectroscopy [23]. This metrological capability may be explicitly exploited in future studies. In the present work, diffraction and spectroscopy are applied primarily as comparative structural probes across facilities and techniques. Beyond this general statement, issues of formal metrological traceability were not pursued further.

Despite this extensive body of work, most studies focus on individual techniques or specific physical questions and do not address the reproducibility and interoperability of quantitative parameters across different measurement platforms, instruments, and laboratories. Systematic comparisons between neutron diffraction, synchrotron X-ray diffraction, X-ray absorption spectroscopy, electrochemical charge extraction, impedance spectroscopy, and DFT-based modeling remain scarce. A noteworthy combination of operando neutron and X-ray diffraction experiments on spinel electrodes was done by Bianchini et al. [24].

To address these challenges, a coordinated interlaboratory study was conducted within the EURAMET OpMetBat (21GRD01) and HyMetBat (24GRD09) projects. A single batch of NMC622 powder and electrode material was distributed to participating laboratories, where independent measurements were performed using high-energy synchrotron XRD, high-resolution neutron diffraction at two neutron sources, Ni, Co, and Mn K-edge XANES and EXAFS spectroscopy, DFT-based electronic structure and XANES simulations, and electrochemical charge extraction combined with *operando* impedance spectroscopy [25].

The objective of this work is to assess the degree of convergence between independent measurement platforms, to identify sources of uncertainty and instrument-to-instrument variability, and to establish traceable links between structural, spectroscopic, electronic, and electrochemical observables. On this basis, a transferable metrological workflow for Ni-rich layered oxide cathode materials is proposed.

## Materials and methods

### Electrodes preparation

Commercial NMC622 powder (BASF) was processed into electrodes at the MEET Battery Center, University of Münster, using in-house industrial-scale mixing, coating, drying, calendaring, and cutting procedures. Positive electrode slurries consisted of NMC622 (95 wt%), carbon black (2 wt%), and PVDF binder (3 wt%) dispersed in *N*-methyl-2-pyrrolidone (NMP). Negative electrode slurries were prepared from graphite and carbon black using carboxymethyl cellulose (CMC) and styrene–butadiene rubber (SBR) binders in aqueous media. Positive electrodes were coated onto aluminum foil, while negative electrodes were coated onto copper foil. The positive electrode areal capacity was adjusted to 2.3 mAh cm⁻^2^. After calendaring, the electrode porosity was approximately 30%. A schematic overview of the electrode manufacturing process is provided in the Supporting Information (Fig. S1).

### Cell assembly and electrochemical assessment

Pouch cells were assembled using a polypropylene separator and a standard electrolyte consisting of 1 M LiPF₆ dissolved in EC:DMC (1:1 by mass). Electrochemical cycling was performed using an IVIUM Vertex potentiostat (100 mA). The lithium extraction level Δ*x* was determined by integrating the charge passed over all cycling steps, assuming an initial lithium content of *x* = 1 in LiₓNi_0.6_Mn_0.2_Co_0.2_O_2_.

For neutron diffraction experiments, NMC electrodes were electrochemically cycled in pouch cells using a graphite-based counter electrode under identical electrolyte and cycling conditions. Pristine reference electrodes (Cell 0) were assembled in the same configuration but kept at open-circuit voltage for the same duration as the cycling experiments.

After completion of cycling, electrodes were disassembled inside an Ar-filled glovebox, washed with pure dimethyl carbonate (DMC), and dried in the glovebox antechamber. The electrodes were then sealed in air-tight containers under Ar atmosphere. Sample mounting for neutron diffraction at SINQ (PSI) was carried out inside an Ar glovebox without air exposure. The electrode material was measured directly on the aluminum current collector foil and sealed in the sample holders using indium wire. Representative cycling curves used for the determination of Δ*x* are provided in the Supporting Information (Fig. S2).

### Crystallographic structure determination with X-rays and neutrons

X-ray powder diffraction (XRPD) measurements were performed at the ID31 beamline at the European Synchrotron Radiation Facility (ESRF), Grenoble, France, through the EU OSCARS program (Momentum Transfer; Zenodo: https://doi.org/10.5281/zenodo.18056756). Data were collected at room temperature using synchrotron radiation with *λ* = 0.1652 Å (≈ 75 keV), corresponding to the high photon energies routinely employed at the ESRF ID31. Instrumental parameters were determined from a LaB_6_ standard and used for subsequent refinements.

Additional synchrotron powder X-ray diffraction data were collected at ESRF beamline BM28 (XMaS, UK CRG, [26]) using monochromatic radiation at an X-ray energy of 10 keV (*λ* = 1.240 Å). The baseline performance of the diffraction setup was verified using a NIST silicon standard (NIST 640) mounted in a 1 mm Kapton capillary. Diffraction patterns were recorded using a Pilatus 300 k detector over a 2*θ* range of 0°–60°, acquired by scanning the detector in 0.5° steps. Standard-based corrections were applied to compensate for small sample-position offsets on the goniometer mount.

Neutron diffraction measurements were performed at the high-resolution powder diffractometer HRPT at the SINQ neutron source (PSI, Switzerland) [27]. Data were collected in high-intensity mode using a neutron wavelength of 1.494 Å at 1 K. Cycled electrodes were transferred into sealed sample holders in an Ar-filled glovebox without air exposure and measured directly on the Al current collector foil.

Time-of-flight neutron powder diffraction data were collected on the high-resolution powder diffractometer (HRPD) at the ISIS Neutron and Muon Source [28], Harwell Science and Innovation Campus, Didcot, UK. Measurements were performed at ambient temperature and pressure. Samples were sealed under argon in a quartz ampule and mounted in a cylindrical vanadium can. Instrument parameters were provided by the ISIS HRPD instrument team.

#### Structure refinement

Rietveld refinements were carried out using the GSAS-II software package [29]. Powder diffraction datasets were refined independently. For refinement of powder samples, thermal displacement parameters of transition metals were constrained equal; for X-ray diffraction refinement, occupancies of transition metals were not refined and kept 0.6, 0.2, and 0.2 for Ni, Mn, and Co, respectively, due to low X-ray scattering contrast. For neutron diffraction patterns, their occupancies were refined independently with sum of all occupancies constrained to 1. For refinements of cycled electrodes, lattice parameters and microstrain were refined independently for each state of charge. Isotropic atomic displacement parameters were constrained to be equal across samples (and, where applicable, across transition metal sites), and all occupancies were constrained except for Li. The Li occupancy of the uncycled electrode was fixed to the value obtained from refinement of the corresponding powder reference measured on the same instrument; Li occupancies for subsequent states of charge were refined without constraints. Complementary X-ray and neutron diffraction refinements were performed using standard-derived instrument parameter files. Where relevant (XMaS), standard-based corrections were applied to remove systematic peak-position offsets caused by sample movement.

### X-ray absorption spectroscopy

Ni, Co, and Mn K-edge X-ray absorption near-edge structure (XANES) and extended X-ray absorption fine structure (EXAFS) measurements were performed on pristine NMC622 cathode material at the Soleil Synchrotron on the Samba beamline [30]. Measurements were conducted at room temperature in transmission mode. The investigated material was LiNi_0.6_Mn_0.2_Co_0.2_O_2_, processed into composite electrodes containing polyvinylidene difluoride (PVDF) binder and carbon black as conductive additive and coated onto 15 μm aluminum foil. The electrode thickness was adjusted to provide an optimal X-ray absorption step of approximately 1.5 at the respective absorption edges. Energy calibration was performed using simultaneous transmission measurements of Ni, Co, and Mn metal reference foils. Quantitative analysis of XANES and EXAFS spectra was carried out using the Demeter (IFEFFIT) software package (compare [31]).

### Calculation of electronic structure

Electronic band structure, total and partial densities of states (TDOS and PDOS), and X-ray absorption spectra were calculated for NMC622 in the rhombohedral $R{\bar{3}}m$ structure using density functional theory (DFT). All electronic structure and spectroscopy calculations were performed using the Quantum ESPRESSO package [32–34]. The electronic structure calculations were carried out using a plane-wave basis set and pseudopotential formalism. Troullier–Martins norm-conserving pseudopotentials were employed together with the spin-polarized generalized gradient approximation (GGA) in the Perdew–Burke–Ernzerhof (PBE) exchange–correlation functional. Strong on-site electron–electron interactions in the transition metal 3d states were treated within the DFT + U framework using the rotationally invariant approach. For NMC622-type layered oxides, typical effective Hubbard parameters reported in the literature are Ueff ≈ 5–6 eV for Ni, 4–5 eV for Mn, and 3–4 eV for Co (for example, [35]). In principle, these values can be refined for a given system by linear-response approaches or by calibration against experimental data or hybrid-DFT calculations. In the present work, values were adopted, which are sufficient to reproduce the experimentally observed oxidation states and XANES line shapes. The effective Hubbard parameters applied were UNi = 5.6 eV, UMn = 2.8 eV, and UCo = 2.5 eV. Additional justification of the selected U values is provided in the Supporting Information. A 5 × 2 × 1 supercell (120 atoms) was constructed from the rhombohedral unit cell. Brillouin-zone sampling employed a 6 × 6 × 6 Monkhorst–Pack grid. Ni, Mn, and Co K-edge XANES spectra were calculated using the XSpectra module of Quantum ESPRESSO, including a core–hole treatment and both dipolar and quadrupolar transitions to enable comparison with experiment. Further computational details are provided in the Supporting Information.

## Results

### Diffraction studies

#### Pristine material

**Figure 1:**
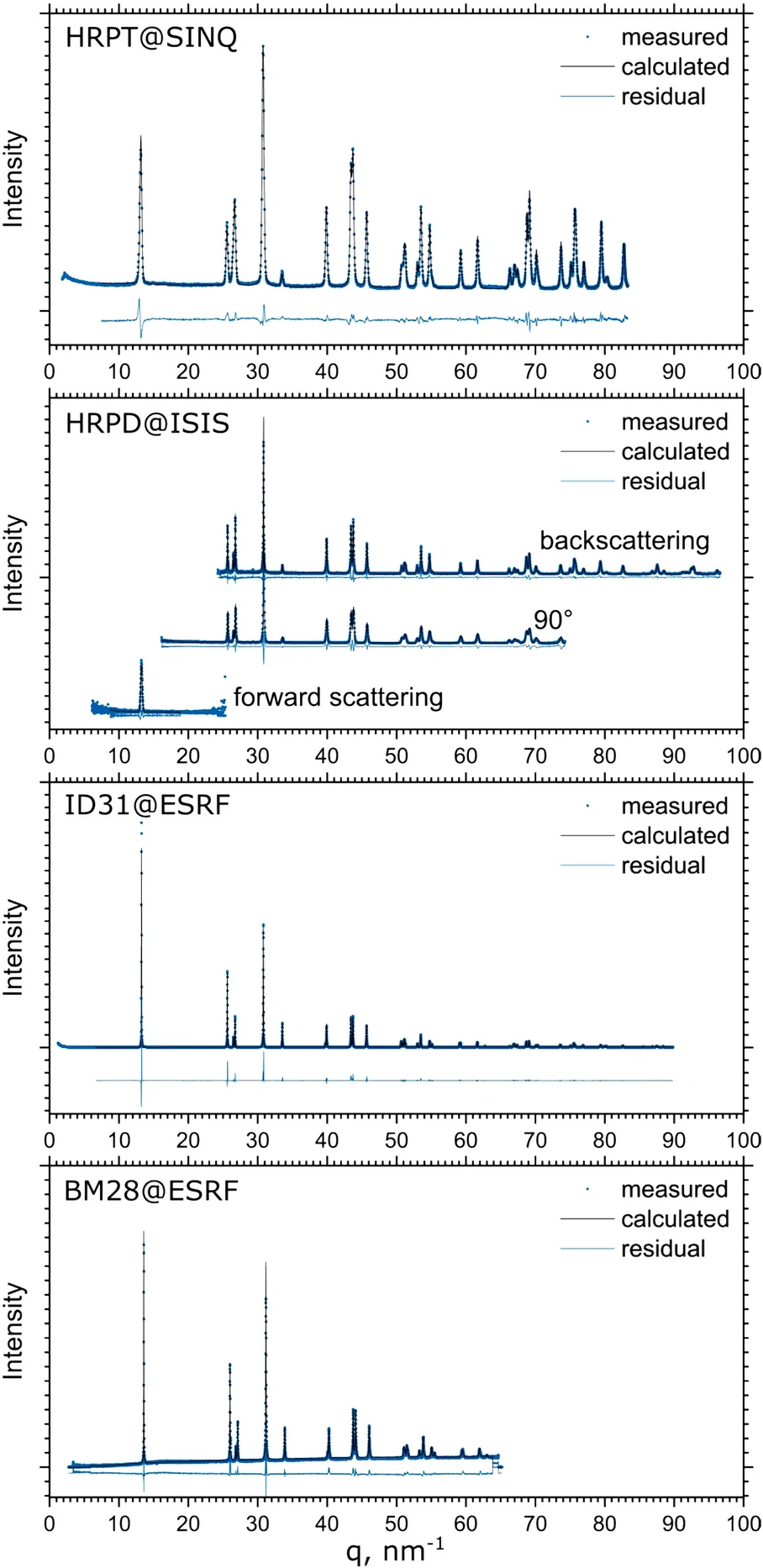
High-resolution diffraction patterns of pristine NMC622. Comparison of synchrotron XRD (ESRF ID31, ESRF XMaS BM28), neutron diffraction from HRPD (ISIS), and neutron diffraction from HRPT (SINQ, 1 K). All patterns show excellent agreement in peak positions and line shapes. Minor thermal contraction in the HRPT data is consistent with cryogenic temperature. Fits and residuals are from GSAS-II Rietveld refinement.

Aliquots of the same pristine NMC powder were analyzed with XRD at ESRF (OSCARS Momentum Transfer) at room temperature at wavelength of 0.1652 Å, XMaS beamline (BM28) at ESRF at wavelength of 1 Å, neutron diffraction at time-of-flight HRPD at ISIS at room temperature, and HRPT diffractometer at SINQ at 1 K temperature at neutron wavelength of 1.494 Å. The diffraction patterns are shown in Fig. 1.

Rietveld refinement was performed independently for all four datasets. The thermal displacement parameters of transition metal (TM) atoms were constrained to be equal within a dataset. For XRD, the Ni, Mn, and Co occupancies were fixed at 0.6, 0.2, and 0.2, respectively. For ND, the transition metal occupancies were constrained to have sum equal to 1. After the refinement, the occupancies of all elements were renormalized so that the maximum occupancy of a single position is equal to 1. The results of the Rietveld refinement are listed in Table 1.

**TABLE 1:** Rietveld-refined crystallographic parameters of LiNi_0.6_Mn_0.2_Co_0.2_O_2_ (NMC622) obtained from synchrotron X-ray powder diffraction (ESRF OSCARS, ESRF XMaS) and neutron powder diffraction measurements at HRPD (ISIS) and HRPT (SINQ).

Parameter	ESRF OSCARS	ESRF XMaS	HRPD@ISIS	HRPT@SINQ
Temp	RT	RT	RT	1 K
λ	0.1652 Å	1 Å	TOF	1.494 Å
a	2.87329 ± 0.00013	2.8674 ± 0.00005	2.86794 ± 0.00006	2.86625 ± 0.00011
c	14.2141 ± 0.0003	14.2185 ± 0.0001	14.21643 ± 0.00025	14.18811 ± 0.00027
Ni occ	0.6	0.6	0.549 ± 0.003	0.542 ± 0.003
Mn occ	0.2	0.2	0.191 ± 0.003	0.191 ± 0.003
Co occ	0.2	0.2	0.164 ± 0.003	0.161 ± 0.003
Li occ	0.96 ± 0.06	0.94 ± 0.05	0.776 ± 0.015	0.754 ± 0.011
O occ	0.861 ± 0.014	1	1.0000 ± 0.0024	1.0000 ± 0.0021
Weighted R-factor	11.28%	10.23%	10.39%	4.55%

X-ray and neutron datasets were refined independently. For all datasets, the isotropic thermal displacement parameters of the transition metal atoms were constrained to be equal. For X-ray diffraction, Ni, Mn, and Co occupancies were fixed to their nominal values, whereas for neutron diffraction the transition metal occupancies were constrained to sum to unity. Occupancies were renormalized after refinement such that the maximum site occupancy equals one. Values in parentheses indicate estimated standard deviations.

The unconstrained refinement strategy is not intended to provide absolute chemical composition, but to probe the intrinsic measurement sensitivity and parameter correlations of different diffraction techniques under realistic battery-relevant conditions.

Due to the low X-ray scattering contrast, Ni, Mn, and Co occupancies were not refined for XRD measurements. The refined occupancies of transition metals obtained with neutron diffraction agree well within the error bars. Besides, the lattice parameters agree well between the XRD and ND at room temperature. For ND at 1 K, a small thermal lattice contraction is observed. The occupancies of TM and lithium are also in good agreement within the error bar for two ND measurements at different temperatures. Both neutron experiments demonstrate the composition TM ratio close to the nominal 6:2:2 ratio. However, the refined occupancies of Li and O differ dramatically from the results of XRD analysis, which is associated with the low sensitivity of high-energy X-rays to light elements such as oxygen and especially lithium. This demonstrates the power of neutron diffraction for a quantitative analysis of lithium content in the battery materials, which is an important aspect for metrology of lithium-based batteries. In general, the results demonstrate gratifying reproducibility and consistency of the results across different instruments and different experimental conditions.

#### Electrode material from cycled cells

**Figure 2:**
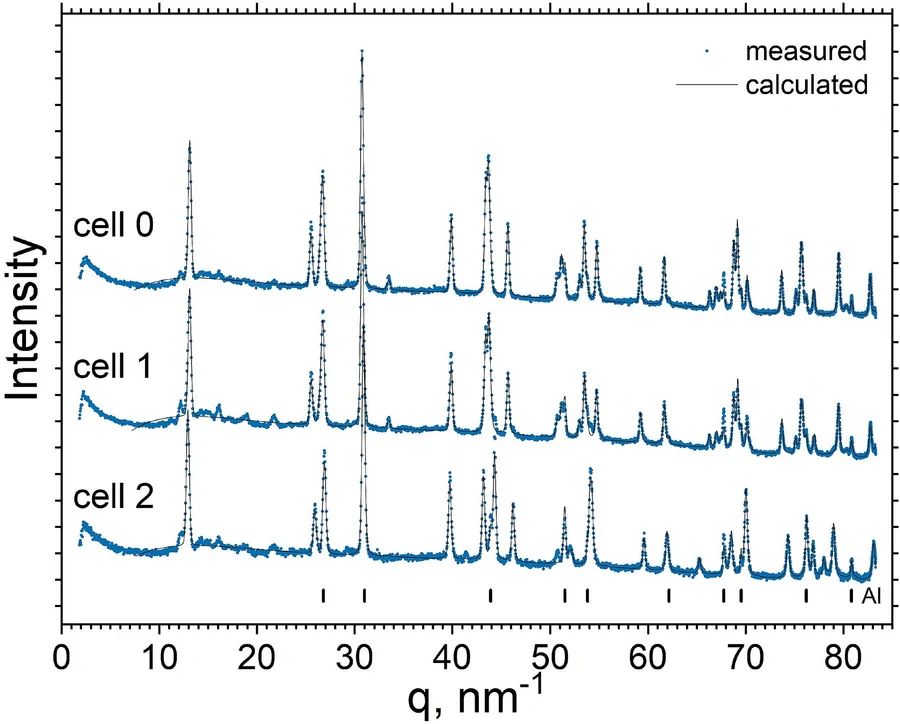
Neutron diffraction patterns of the cycled NMC electrodes. The graphs are offset along vertical axis. The positions of the peaks corresponding to Al foil are marked at the bottom. All other calculated peaks are attributed to NMC.

To study the quantitative capabilities of the neutron diffraction in measuring the lithium content in the cathode material, we measured the neutron diffraction of the pristine and charged (delithiated) electrodes. Electrodes were charged to a low (~ 5%), where no significant structural changes are expected, and high (~ 50%) state of charge. The diffraction patterns, measured from the charged electrodes, are shown in Fig. 2. The patterns demonstrate a slight peak shift, associated with the lattice expansion. The lattice parameters of NMC change non-monotonically with charging and are studied in great details [36, 37]; our observations (Table 2) agree well with their reported results. The diffraction patterns of charged cells demonstrate some extra peaks (for example, around 20 nm^−1^) which can be attributed to SEI (solid electrolyte interface) components or components of the electrolyte.

**TABLE 2:** Lattice parameters, refined lithium occupancies, and relative changes in lithium content in Li_x_Ni_0.6_Mn_0.2_Co_0.2_O_2_ derived from neutron powder diffraction (ND) and electrochemical (EC) measurements.

Sample	a, Å	c, Å	Li occupancy from ND	Δx(Li), from ND	Δx(Li), from EC
Cell 0	2.866	14.181	0.731	-	-
Cell 1	2.864	14.193	0.651 ± 0.018	8.0% ± 1.8%	6.5%
Cell 2	2.826	14.405	0.471 ± 0.023	26.0% ± 2.3%	41.2%

Lithium occupancies and changes in lithium content were obtained from Rietveld refinement of neutron diffraction patterns, while electrochemical values were calculated from the corresponding cycling curves. Values in parentheses indicate estimated standard deviations.

Table 2 lists the results of refinement of Li occupancy and the change of lithium content in Li_x_Ni_0.6_Mn_0.2_Co_0.2_O_2_, calculated from the Rietveld refinement of the diffraction patterns and from the electrochemical cycling curves.

The refinement of Li content agrees well with the EC-derived for both samples. The occupancy of the lithium positions is highly positively correlated with the thermal displacement parameter in the Rietveld refinement procedure (covariance = 0.590, 0.581 and 0.569 in the samples, respectively); at the same time, they are also correlated in a physical world, where removal of Li causes lattice expansion and hence, increased thermal displacement parameter. For this reason, the accurate quantitative refinement of lithium content would require some prior knowledge about the relations between lithium content and the structure of the material.

### XANES and EXAFS

#### Ni, Co, and Mn K-edge EXAFS analysis

**Figure 3:**
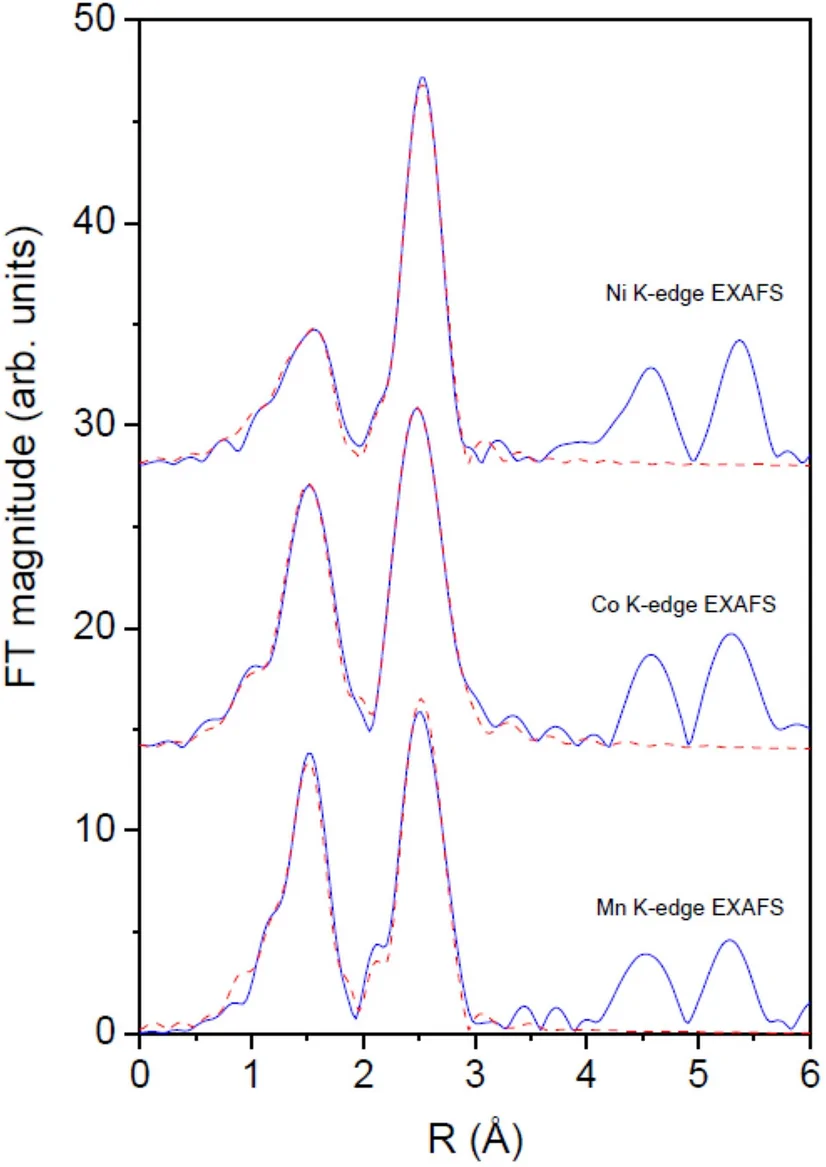
EXAFS**.** Fourier transform magnitude of k^3^-weighted Ni, Co, and Mn K-edge EXAFS spectra of the pristine NMC622 cathode material calculated in the k range of 3–15 Å^−1^, in case of Ni and Mn spectra, and in the k range of 3–12 Å^−1^ in case of Co spectrum: Experiment – (blue solid line); best fit EXAFS model calculated in the R range of 0.9 to 3.0 Å—(red dashed line), showing nearest neighbor metal–oxygen and metal–metal coordination shells. Coordination distances agree with ND/XRD.

Ni, Co and Mn K-edge EXAFS analysis is used to detect local structure of transition metal (TM) cations (Ni, Co and Mn) in the pristine NMC622 cathode material. In Fourier transform magnitude of the EXAFS spectra (Fig. 3), the contributions of photoelectron scattering on the nearest shells of neighbors around Ni, Co, and Mn atoms are observed in the R range up to about 6 Å. In quantitative EXAFS analysis of each EXAFS spectrum, a FEFF model [23] is constructed, based on crystal structure of NMC622 with space group R-3m with the lattice constant *a* = *b* = 2.87189(4) Å, *c* = 14.2233(1) Å, *α* = *β* = 90°, *γ* = 120° [12], where Ni, Co, and Mn cations are coordinated to 6 oxygen atoms at a distance of 1.92 Å, 6 TM atoms at 2.87 Å (with the relative occupancies: Ni 60%, Co and Mn 20%), and 6 Li atoms at 2.89 Å (see Fig. S4 form Ref. [12]). (Contributions of more distant coordination shells, observed in FT EXAFS spectra in the R range between 3 and 6 Å, are not included in the model.) The FEFF model of these nearest coordination shells comprised five single scattering paths up to 3 Å, with 8 variable parameters: coordination shell distance (R) and Debye–Waller factors (*σ*2) of all single scattering paths, and the amplitude reduction factor S02 and the shift of energy origin of the photoelectron ΔEo, common to all scattering paths, are introduced.

The shell coordination numbers were fixed to the crystallographic values, considering the relative occupancies of TM cations in the crystal structure. The Debye–Waller factors of TM neighbors are constrained to the same value, reducing uncertainties due to strong correlations between these parameters. A very good agreement between the model and the experimental spectra is found using the *k* range of 3–15 Å^−1^ in case of Ni and Mn spectra, and the *k* range of 3–12 Å^−1^ in case of Co spectrum, and the *R* range of 0.9–3.0 Å. Best fit results from Fig. 3 are presented in Tables 3–5 in supporting information.

The results of local structure around Ni, Co, and Mn cations in pristine NMC622 material are in good agreement with XRD data on this material, and with EXAFS results reported for the NMC622 fresh material in Ref. [12], except for one minor difference in TM–TM interatomic distances. The results are consistent with the XRD data, measured on the same material: although the diffraction does not distinguish between TM atoms, weighted average of the TM-O distances derived from EXAFS (0.6 × 1.972 + 0.2 × 1.919 + 0.2 × 1912 = 1.949 Å) agrees well with the distances from the refinement of the structure from diffraction data (1.962 Å). The TM-O distance, obtained by the Rietveld refinement of diffraction patterns (XRD and ND at room temperature) is close to the weighted average of TM-O distances.

Our EXAFS results indicate that the Mn–Mn and Co–Mn distance in the crystal structure are slightly longer than Ni–Mn, Ni–Co, and Ni–Ni distances.

In comparison with the diffraction data, EXAFS provides similar values of the thermal displacement (Debye–Waller factor) parameters. The transition metal—oxygen distance derived from structures obtained by Rietveld refinement of diffraction patterns is 1.962 Å, which is in good agreement with weighted average of the distances obtained from EXAFS (0.6 × 1.972 + 0.2 × 1.919 + 0.2 × 1.912 = 1.949 Å), indicating that the results of different techniques are structurally consistent.

#### Ni, Co, and Mn K-edge XANES analysis

**Figure 4:**
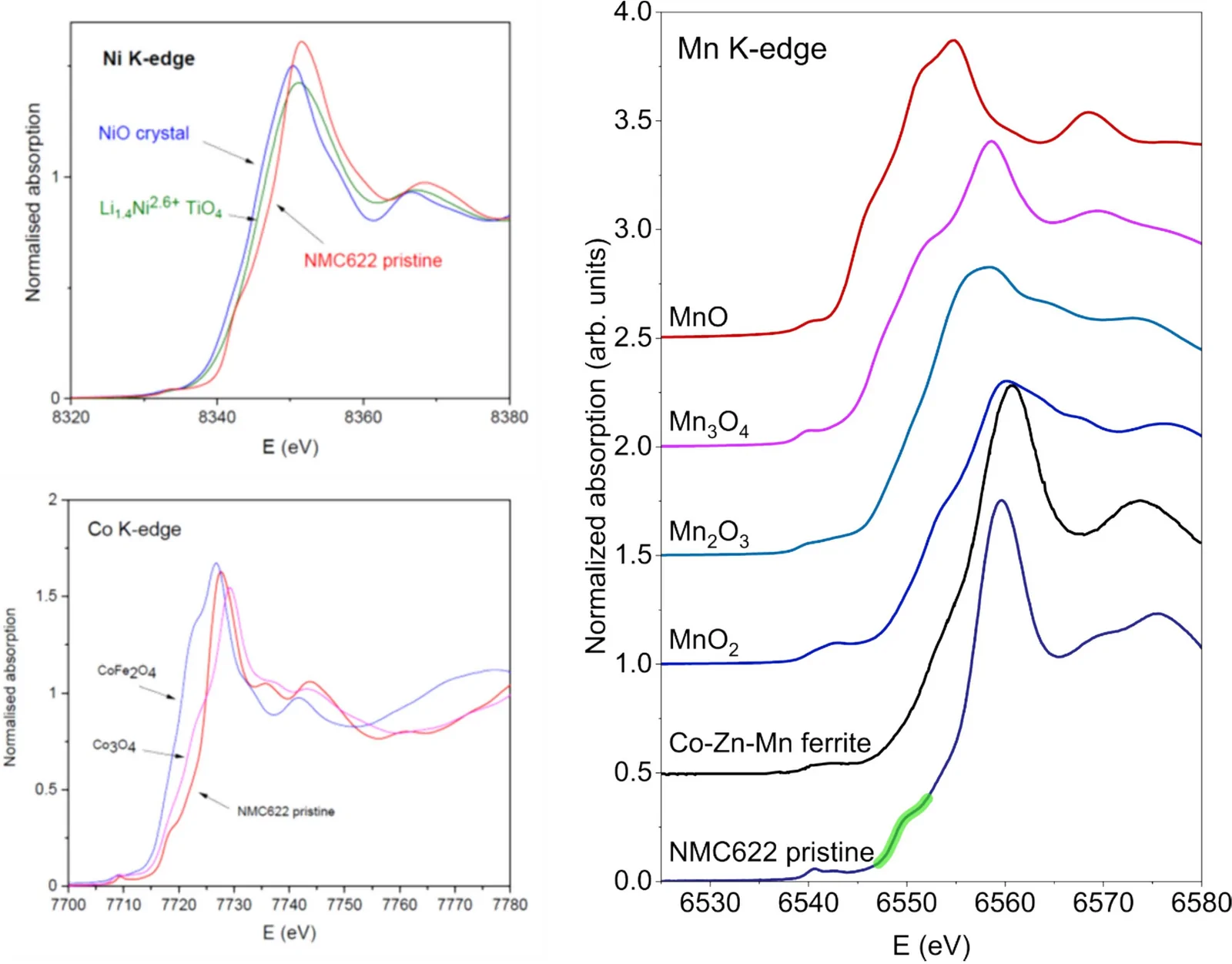
Experimental transition metal K-edge XANES spectra of NMC622. (a) Ni K-edge, normalized Ni K-edge XANES spectra measured on NMC622 pristine sample at ESRF. The spectrum of crystalline NiO, as a reference for Ni^2+^, and Li_1.4_NiTiO_4_ cathode material, as reference for Ni^2.6+^ , are shown for comparison. (b) Co K-edge, normalized Co K-edge XANES spectra measured on NMC622 pristine sample. The spectrum of crystalline CoFe_2_O_4_, as a reference for Co^2+^, and Co_3_O_4_, as reference for Co^2.7+^, are shown for comparison. (c) Mn K-edge, normalized Mn K-edge XANES spectra measured on NMC622 pristine sample. Spectra of reference Mn compounds with different Mn valence states between Mn^2+^ and Mn^4+^ (MnO, Mn_3_O_4_, Mn_2_O_3_, MnO_2_, and quaternary Ferrite samples Co-Zn-Mn^4+^ [X2] are shown for comparison.

**Figure 5:**
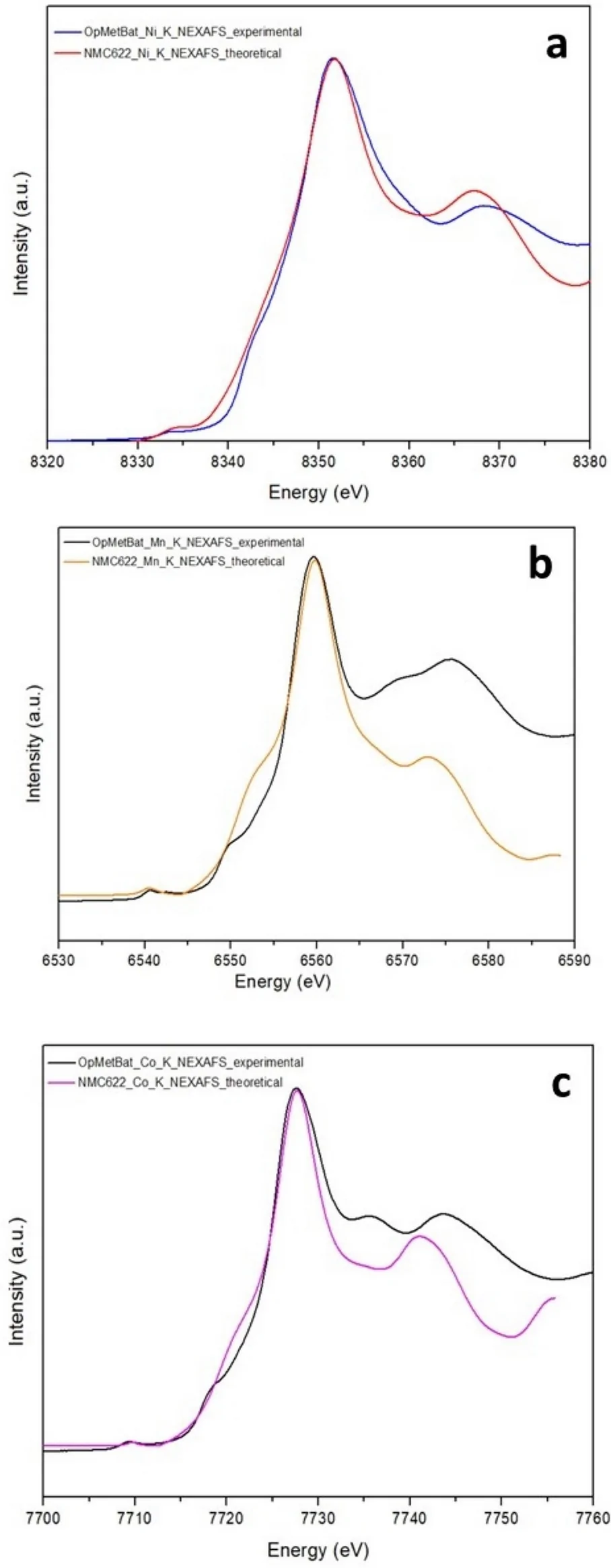
Comparison of experimental and calculated XANES at K-edges of (a) Ni (b) Mn (c) Co.

**Figure 6:**
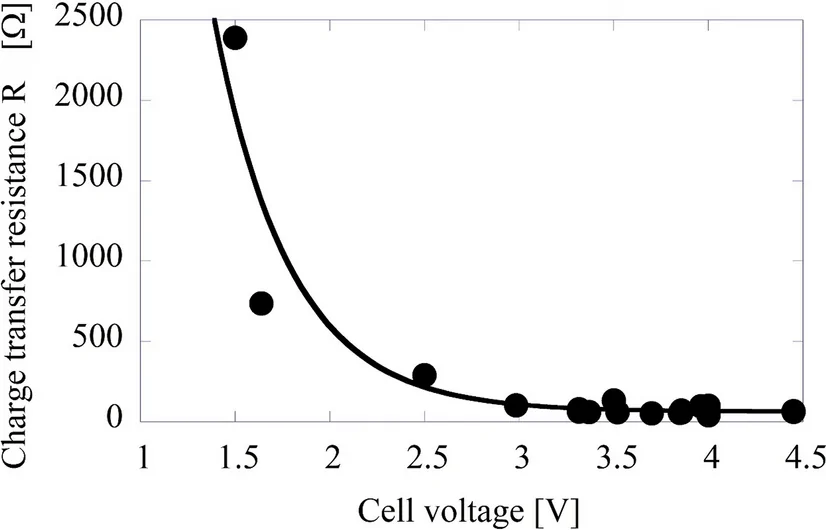
Change of the charge-transfer resistance R during change of voltage, obtained from RCT from quantitative impedance analysis.

The Ni, Co and Mn K-edge XANES analysis is used to monitor valence and local symmetry of Ni, Co and Mn cations in the pristine NMC622 cathode material. Normalized XANES spectra together with the spectra of corresponding Ni, Co and Mn reference compounds are shown on Figs. 4, 5 and 6. Different local environments of the metal cation result in different K-edge profiles in the XANES spectra, and the K-edge energy position is correlated with the valence state of the metal cations in the sample [38–41].

The average valence state of Ni cations in NMC622 pristine material is estimated from the Ni K-edge energy position (Fig. 4). Comparison with Ni K-edge XANES spectra of the reference compounds (crystalline NiO with octahedrally coordinated Ni^2+^ cations, and crystalline Li_1.4_NiTiO_4_ cathode material for Li-Ion battery with 40% Ni^2+^ and 60% Ni^3+^ cations with octahedral oxygen coordination [39]) shows that the energy of the Ni K-edge is shifted to higher energies by approximately 1.5 eV between Ni^2+^ and Ni^3+^ valence state Refs. [39, 40]. The Ni K-edge energy position of NMC622 pristine sample indicates that the sample contains the Ni predominantly as Ni^3+^ cations with an average Ni valence of 3.0 ± 0.2. The results of the Ni XANES results reported for the NMC622 fresh material in Ref. [40] indicate average Ni valence in fresh material to be 2.7 + .

The average valence state of Co cations in NMC622 pristine material is estimated from the Co K-edge energy position (Fig. 5). For Co cations coordinated to oxygen, a shift of about 3 eV per valence state has been established [38]. Comparison with Co K-edge XANES spectra of the reference compounds with different Co valence states (crystalline CoFe_2_O_4_, as a reference for Co^2+^, and Co_3_O_4_ as reference for Co^2.7+^ [38]) shows that average Co valence state in NMC622 pristine sample is about 3.0 ± 0.2). This is in agreement with the Co XANES results reported for the NMC622 fresh material in Ref. [40].

The average valence state of Mn cations in NMC622 pristine material is estimated from the Mn K-edge energy position (Fig. 6). For manganese atoms coordinated with oxygen, a shift of 3.3 eV per valence state has been established [38, 39, 41]. Comparison with Mn K-edge XANES spectra of the reference compounds with different Mn valence states between Mn^2+^ and Mn^4+^ (MnO, Mn_3_O_4_, Mn_2_O_3_, MnO_2_ , and quaternary Ferrite samples Co–Zn–Mn^4+^ [38]) shows that the Mn K-edge energy position of NMC622 pristine sample coincides with the edge position of the quaternary ferrite sample Co–Zn–Mn, which indicates that the Mn in the sample comes predominantly as Mn^4+^ cations (average Mn valence 4.0 ± 0.2). This is in agreement with the Mn XANES results reported for the NMC622 fresh material in Ref. [40].

### Density of states and comparison of experimental and theoretically calculated Ni/Mn/Co K-edge XANES

To understand the physical properties of material, the electronic density of states (DOS) plays an important role due to providing simply way to characterize electronic structure of materials. The calculated lattice constant is as follows: *a* = *b* = 2.6997(0) Å, *c* = 14.2777(0) Å, *α* = *β* = 90°, *γ* = 120°, where Ni, Co, and Mn cations are coordinated to 6 oxygen atoms at a distance of 1.95 (3) Å (with the occupancies: Ni 60%, Mn and Co 20%). Figure 5 compares experimental and calculated Ni, Mn, and Co K-edge XANES spectra. The calculations reproduce the principal spectral features and edge positions. The agreement supports the crystallographic model derived from diffraction and is consistent with the oxidation-state assignments obtained from XANES analysis.

### Electrochemical investigation

The electrode was cycled in the operando cell detailed in Ref. [42]. Figure S12 shows a representative cycling curve obtained with a charge and discharge current density of 0.5 mA/cm^2^ and voltage range from 0.2 V to 4.3 V. The charging time range was set to 10,000 s and the discharge time range to 50,000 s.

As shown in Fig. 6, the charge-transfer resistance decreases strongly with increasing cell voltage and approaches a nearly constant low value above approximately 3 V. This behavior demonstrates the pronounced voltage dependence of the electrochemical kinetics in the NMC622 electrode. The result further illustrates that impedance spectroscopy provides a sensitive functional observable complementary to diffraction- and spectroscopy-based structural characterization.

The strong decrease of *R*_ct_ with increasing cell voltage is qualitatively consistent with the voltage-dependent evolution of the Ni–O electronic structure in NMC622. Operando Ni L-edge X-ray Raman spectroscopy on related NMC622 electrodes showed that higher cell voltages are associated with an increased Ni^3+^ contribution in the bulk electronic structure [43]. In the present work, DFT calculations indicate substantial hybridized Ni(3d)–O(2p) contributions near the Fermi level. Since electrochemical charge-transfer processes involve electronic states in the vicinity of the Fermi level, the observed voltage dependence of *R*_ct_ is consistent with increased electrochemical accessibility of the Ni–O redox network during delithiation.

## Discussion

Diffraction patterns obtained from synchrotron X-ray diffraction at ESRF and neutron diffraction at both ISIS and SINQ show excellent mutual agreement for pristine NMC622, establishing a consistent structural reference state. Only minor thermal contraction is observed at 1 K, while refined transition metal occupancies derived from neutron diffraction match the nominal stoichiometric ratios within ± 0.003. As expected, high-energy X-ray diffraction yields unreliable oxygen occupancies due to its intrinsically low sensitivity to light elements, underscoring the complementary roles of X-ray and neutron probes already at the level of pristine material.

A key outcome of this study is the close agreement of lattice parameters and transition metal occupancies obtained from two independent neutron facilities. The measured lattice parameters for pristine NMC622 differ by less than Δ*a* = 0.006 Å and Δ*c* = 0.028 Å between HRPD and HRPT after correction for temperature differences, values that lie well within the combined instrumental uncertainties of the respective diffractometers. These results demonstrate that the measurement model—including instrument profile functions, wavelength calibration, and background treatment—is sufficiently robust to yield harmonized results across platforms.

The refined transition metal occupancies (Ni:Mn:Co ≈ 0.55:0.19:0.16) are consistent between the ISIS and SINQ neutron diffraction measurements, with relative deviations below 1.5%. This level of agreement is particularly notable given the substantial differences in measurement principle (time-of-flight versus monochromatic diffraction), instrument geometry and resolution, sample environment (aluminum-foil-supported electrodes versus powder mounts at different temperatures), data reduction pipelines, and operator practices. Together, these observations demonstrate that neutron diffraction can provide interoperable and reproducible quantification of atomic occupancies, a prerequisite for metrological traceability in battery materials.

Traceability of the refined lithium occupancies is established via calibrated neutron wavelength standards, instrument profile functions derived from certified reference materials (e.g., vanadium, Si, LaB₆), and cross-validation against coulometric charge extraction, providing an SI-linked measurement chain from diffraction intensities to lithium stoichiometry. In metrological terms, the traceability chain links measured neutron intensities to absolute structure factors via instrument calibration with certified reference materials, from which site occupancies are refined and converted into lithium stoichiometry. This stoichiometry is independently linked to the SI through coulometric charge extraction, providing an unbroken measurement chain from diffraction counts to charge in coulombs.

Lithium occupancy emerges as the parameter of primary metrological interest when linking structural and electrochemical descriptions. The neutron-derived Li occupancies for pristine material (0.78–0.76) exhibit internal consistency across instruments, whereas the XRD-derived value (0.96 ± 0.06) reflects the intrinsic insensitivity of high-energy X-ray diffraction to lithium. Under these conditions, XRD cannot be considered a traceable technique for quantitative determination of Li content.

In cycled electrodes, neutron-derived Δ*x*(Li) values agree with electrochemically extracted charge within the combined uncertainty of both methods for moderate degrees of delithiation. At deeper delithiation, the systematic deviation (ND < electrochemistry) is consistent with well-documented parasitic reactions like trace water decomposition or formation of passivating layers on cathode and anode, and surface-related processes in Ni-rich layered cathodes [44]. Neutron diffraction thus measures the physically meaningful bulk lithium occupancy, whereas electrochemical methods integrate all faradaic and non-faradaic contributions. This distinction is essential for establishing a metrological chain linking electrochemical charge to crystallographic state variables. However, the refined lithium occupancies correlate with thermal displacement parameter of lithium, which is also a function of lithium content due to lattice expansion of NMC material during charge. This aspect contributes to inability to accurately refine lithium occupancies based exclusively on the diffraction experiments. A significant fraction of the discrepancy might be attributed to this phenomenon. Therefore, using complementary techniques, providing similar information is necessary for accurate determination of defect concentration and state of charge.

Sensitivity to light elements further differentiates the measurement capabilities of neutron and X-ray diffraction. Oxygen occupancies refined from neutron diffraction remain unity within ± 0.002, while high-energy X-ray diffraction yields artificially reduced values. This behavior reflects parameter correlation effects in Rietveld refinement, where weak scattering contrast, background modeling, and atomic displacement parameters interact. Neutron diffraction therefore provides the appropriate measurement capability for determining oxygen stoichiometry with quantified confidence.

Spectroscopic measurements provide an independent constraint on the local atomic environment and enable cross-validation of diffraction-derived structural models. Ni, Co, and Mn K-edge XANES and EXAFS results yield oxidation states of Ni^3^⁺, Co^3^⁺, and Mn^4^⁺, and the refined local coordination distances closely match those obtained from neutron and X-ray diffraction. The absence of systematic energy drift in the XANES measurements, with reproducibility better than ± 0.01 eV, further supports the stability and reliability of the spectroscopic data as a secondary anchor point within the metrological framework.

An anonymous reviewer has drawn attention to a weak step-like feature in the Mn K-edge XANES spectrum of NMC622 in the energy range 6548–6553 eV (Fig. 6, green marker). This observation is noteworthy because, when plotted relative to the absorption edge, the feature resembles a broadened onset of absorption and invites comparison with electronic states appearing near the Fermi level in other spectroscopies.

Such an analogy should not be interpreted as evidence for metallic conductivity. The Mn K-edge probes element-specific 1s → 4p transitions, while the corresponding final states are strongly hybridized with oxygen and neighboring transition metal states. In layered NMC materials, electronic states near the Fermi level are generally associated with hybridized Ni–O bands rather than Mn-centered states.

The observed feature may therefore reflect the influence of delocalized transition metal–oxygen electronic states on the absorption onset. While the present data do not permit a definitive assignment, the observation illustrates how element-specific XANES can provide sensitivity to collective electronic structure effects extending beyond the nominal absorbing atom. A more detailed investigation combining density-of-states calculations, photoelectron spectroscopy, and resonant X-ray spectroscopies is beyond the scope of the present work.

Consistency between experiment and theory provides an additional level of validation. The agreement between experimentally measured and DFT-simulated XANES spectra confirms that the structural models derived from diffraction are self-consistent across different physical observables. This coherence strengthens the conclusion that the combined neutron diffraction → DFT → XANES workflow yields a transferable and internally consistent description of the electronic structure.

Finally, operando impedance spectroscopy reveals systematic variations in charge-transfer resistance that correlate with neutron-detected changes in lattice parameters and lithium content. The observed increase in resistance at deep delithiation and its recovery upon discharge suggest that impedance metrics may serve as indirect, yet quantifiable, indicators of structural state. In this context, the links between voltage–capacity features and impedance response, and their dependence on charge movement rate and protocol parameters, have recently been discussed from a metrology perspective [45].

This correlation points toward the potential development of traceable electrochemical indicators for state-of-charge and state-of-health determination. In parallel, data-driven SOH estimation methods increasingly exploit features extracted from routinely recorded voltage/current data during cycling or charging. Recent work [46] demonstrates that advanced feature extraction combined with optimized regression models can yield high-accuracy SOH predictions across multiple public datasets.

Neutron diffraction provides the quantitative lithium inventory; electrochemical charge extraction provides the electrochemical reference; impedance spectroscopy provides the functional transport response; XANES/EXAFS and DFT provide electronic structure consistency checks.

Taken together, these results demonstrate that neutron diffraction provides the highest measurement capability for quantitative determination of lithium occupancy and transition metal stoichiometry, while synchrotron X-ray diffraction delivers highly traceable lattice parameters but lacks sufficient sensitivity to light-element composition. X-ray absorption spectroscopy, in combination with density functional theory, supplies complementary spectroscopic and electronic constraints that reinforce the diffraction-based structural interpretation. The observed agreement across multiple facilities and techniques supports the development of transferable reference workflows and interlaboratory comparison protocols aimed at metrological standardization of battery materials.

## Conclusions

This coordinated multi-instrument study provides a metrological assessment of LiNi_0.6_Mn_0.2_Co_0.2_O_2_ (NMC622) as a candidate reference material for interlaboratory structural measurements. Neutron diffraction is shown to deliver reproducible and traceable structural parameters across independent facilities, with excellent agreement in lattice parameters and transition metal occupancies despite differences in instrument type, resolution function, and operator practice. This robustness demonstrates that the neutron diffraction measurement model is suitable for metrological applications.

Lithium occupancies derived from neutron diffraction are internally consistent and quantitatively aligned with electrochemical charge extraction at low-to-moderate states of delithiation, establishing a traceable link between structural and electrochemical observables. In contrast, synchrotron X-ray diffraction provides highly precise and traceable lattice parameters but lacks the sensitivity required for quantitative determination of light elements such as lithium and oxygen. Neutron diffraction therefore represents the appropriate reference technique for stoichiometric analysis of these species.

X-ray absorption spectroscopy (XANES/EXAFS) supplies independent, element-specific information on local coordination environments and oxidation states, which is fully consistent with the diffraction-derived structural models. Density functional theory simulations reproduce both the electronic structure and XANES line shapes, confirming the internal consistency of the combined experimental–computational framework. Operando impedance spectroscopy captures state-dependent changes in charge transport and correlates with structural evolution during cycling, highlighting its potential role in future metrological workflows for state-of-charge and state-of-health assessment.

Laboratory X-ray diffraction remains valuable for initial phase identification and qualitative tracking of lattice changes during (de)lithiation, while synchrotron radiation significantly improves statistical quality and refinement stability. The most substantial improvement in quantitative structural refinement, however, is achieved with neutron diffraction, which enables accurate determination of site occupancies for all elements, including lithium and oxygen. The near-identical results obtained from neutron diffraction experiments performed independently at SINQ and ISIS underscore the high level of interfacility reproducibility, a result that cannot be assumed a priori and is not always observed in round-robin comparisons across scientific disciplines [47].

Finally, the close agreement between lithium occupancies derived from neutron diffraction and coulometric analysis demonstrates the quantitative reliability of the structural measurements (compare [5]). Deviations observed after extended cycling can be attributed to parasitic side reactions and degradation processes that do not contribute to reversible charge storage, providing additional diagnostic insight into cell performance. Taken together, the results establish a coherent and transferable multi-method reference workflow and demonstrate the feasibility of using NMC622 as a candidate reference material for interlaboratory comparison campaigns in battery materials metrology.

## Supplementary Information

Below is the link to the electronic supplementary material.

Supplementary file1 (DOCX 6676 kb)

## Data Availability

Data are available from the corresponding authors on request.

## Abbreviations

AOP: Advanced oxidation processes
BM28 (XMaS): X-ray and magnetic scattering beamline at ESRF
CMC: Carboxymethyl cellulose
CSCS: Swiss National Supercomputing Center
DFT: Density functional theory
DFT + U: Density functional theory with Hubbard U correction
DMC: Dimethyl carbonate
DOS: Density of states
EC: Ethylene carbonate
EC (electrochem.): Electrochemical
EF: Fermi energy
ESRF: European Synchrotron Radiation Facility
EXAFS: Extended X-ray absorption fine structure
FEFF: Ab initio multiple-scattering code for EXAFS/XANES
GGA: Generalized gradient approximation
GSAS-II: General structure analysis system II
HPC: High-performance computing
HRPD: High-resolution powder diffractometer (ISIS)
HRPT: High-resolution powder diffractometer for thermal neutrons (SINQ)
ISIS: ISIS Neutron and Muon Source
K-edge: Core-level absorption edge corresponding to K shell
LiPF₆: Lithium hexafluorophosphate
MEET: Münster electrochemical energy technology
ND: Neutron diffraction
NEXAFS: Near-edge X-ray absorption fine structure
NMC: Nickel manganese cobalt oxide
NMC622: LiNi_0.6_Mn_0.2_Co_0.2_O_2_
NMP: *N*-Methyl-2-pyrrolidone
OSCARS: Open Science Clusters’ Action for Research and Society
PDOS: Partial density of states
PBE: Perdew–Burke–Ernzerhof exchange–correlation functional
PVDF: Poly(vinylidene fluoride)
PXRD / XRPD: Powder X-ray diffraction
QE: Quantum ESPRESSO
Rietveld: Rietveld refinement method
RT: Room temperature
SEI: Solid electrolyte interphase
SINQ: Swiss spallation neutron source
SOC: State of charge
SOH: State of health
SBR: Styrene–butadiene rubber
STFC: Science and technology facilities council
TDOS: Total density of states
TM: Transition metal
TOF: Time of flight
TRUBA: TÜBİTAK ULAKBİM High Performance Computing Center
U (Hubbard U): On-site Coulomb interaction parameter
XANES: X-ray absorption near-edge structure
XAS: X-ray absorption spectroscopy
XmaS: X-ray and magnetic scattering beamline (BM28, ESRF)
XRD: X-ray diffraction

## Code availability

Software application or custom code.
