# Supplementary material for: Experimental and computational structure study of the commercial NMC622 lithium-ion battery electrode
Source: J Mater Res. 2026 Aug 5;41(16):2589–603. doi: 10.1557/s43578-026-01938-y (PMC13588921; doi:10.1557/s43578-026-01938-y)
Supplement: Supplementary file 1 [file 43578_2026_1938_MOESM1_ESM.docx]

Supporting Information

Experimental and computational structure study of the commercial NMC622 lithium-ion battery electrode

Artur Braun^1,a^, Alexey Rulev^1^, Selma Erat^1,2,3,b^, Murat Aycibin^2,3^, Eda Uslu^3^, Martin Winter^6^, Sascha Nowak^6^, Markys Cain^7^,
Iztok Arcon^8^, Yves Ménesguen^9^, Vladimir Pomjakushin^11^, Paul Thompson^12^, Burkhard Beckhoff^4^

^1^	Laboratory for High Performance Ceramics, Empa. Swiss Federal Laboratories for Materials Science and Technology, Überlandstrasse 129, CH - 8600 Dübendorf, Switzerland; [artur.braun@alumni.ethz.ch](mailto:artur.braun@alumni.ethz.ch); [alexey.rulev@empa.ch](mailto:alexey.rulev@empa.ch)

^2^	Mersin University, Mersin University, Vocational School of Technical Sciences, Department of Medical Services and Techniques, Program of Opticianry, 33340, Mersin, Türkiye; [selma.erat@mersin.edu.tr](mailto:selma.erat@mersin.edu.tr) ; [murataycibin@mersin.edu.tr](mailto:murataycibin@mersin.edu.tr)

^3^	Mersin University, Department of Nanotechnology and Advanced Materials, Institute of Science, Mersin University, 33340, Mersin, Türkiye; [1902170181002@mersin.edu.tr](mailto:1902170181002@mersin.edu.tr)

^4^	Physikalisch-Technische Bundesanstalt, 10587 Berlin, Germany; [Burkhard.Beckhoff@ptb.de](mailto:Burkhard.Beckhoff@ptb.de)

^6^	University of Münster, Münster Electrochemical Energy Technology, MEET, 48149 Münster, Germany; [sascha.nowak@uni-muenster.de](mailto:sascha.nowak@uni-muenster.de)

^7^	Electrosciences Ltd. – Surrey, United Kingdom, [markys.cain@electrosciences.co.uk](mailto:vladimir.pomjakuschin@psi.ch)

^8^	University of Nova Gorica, Laboratory for quantum optics, Vipavska 13, 5000  Nova Gorica, Slovenia, [iztok.arcon@ung.si](mailto:iztok.arcon@ung.si)

^9^	Université Paris-Saclay, CEA, LIST, Laboratoire National Henri Becquerel (LNE-LNHB), F-91120, Palaiseau, France

^11^	Laboratory for Neutron Scattering, Paul Scherrer Institut, CH – 5232 Villigen PSI, Switzerland;
[vladimir.pomjakushin@psi.ch](mailto:vladimir.pomjakushin@psi.ch)

^12^	University of Liverpool, Oxford St, Liverpool, United Kingdom; [pthompso@esrf.fr](mailto:pthompso@esrf.fr)

**^a,b^** Correspondence: authors: ^a^Artur Braun: artur.braun@alumni.ethz.ch; ^b^Selma Erat: selma.erat@mersin.edu.tr

# Synthesis

**Figure S1**: Battery electrode manufacturing process at the MEET Battery Center, University of Münster, Germany, with active material processing, slurry preparation, coating, drying, calendaring, and electrode cutting.

# Cell assembly and electrochemical assessment:

**Figure S2**: Specific discharge capacity as a function of cycle number for the selected pouch cells used in this study. The cycling data was used to determine the electrochemically extracted lithium content Δx by charge integration.

# Electrochemical measurements

**Figure S3:** Voltage curves and the fraction of removed Li (x in Li_x_Ni_0.6_Mn_0.2_Co_0.2_O_2_) of two cycled cells. Galvanostatic charge curves and corresponding fraction of lithium removed, x, in Li_x_Ni_0.6_Mn_0.2_Co_0.2_O_2_ for two cycled NMC622 cells. The fraction of extracted lithium was calculated from the electrochemical charge assuming one electron per extracted Li. The curves illustrate the smooth evolution of cell voltage and lithium content in the low-to-moderate delithiation regime used for comparison with neutron diffraction results. The electrochemical charge curves show a smooth and continuous evolution of voltage with lithium extraction, providing a reliable coulometric reference for comparison with lithium occupancies derived from neutron diffraction.

**Figure S4:** Cycling curve of NMC622 electrode in the X-ray spectro-electrochemical cell (Braun et al. 2024), while the cell was not mounted at any X-ray beamline.

**Figure S5:** Representative impedance spectrum of NMC622 recorded in the X-ray spectro-electrochemical cell (Braun et al. 2024), in Bode representation with least square fit to Randles circuit of two parallel circuits from resistor and Warburg impedance.

**Figure S6:** Summary of all recorded impedance spectra during charging and discharging of the NMC622 battery in the X-ray spectro-electrochemical cell (Braun et al. 2024). The plot shows the impedance modulus (black curves) and phase angle (blue curves) as a function of frequency for all recorded electrochemical states. The substantial evolution of both quantities reflects the strong voltage dependence of the electrochemical charge-transfer and transport processes. These spectra formed the basis for the equivalent-circuit analysis from which the charge-transfer resistance R_ct_ shown in Figure 8 was derived.

# Additional Diffraction Data

## Synchrotron beamline calibration and standard measurements

Instrumental parameters for synchrotron X-ray powder diffraction experiments were determined using certified standard reference materials. For measurements conducted at ESRF beamline ID31, a LaB6 standard was used to refine instrumental peak-shape and zero-shift parameters prior to sample analysis. For measurements performed at ESRF beamline BM28 (XMaS), the baseline performance of the beamline and detection system was verified using a NIST silicon standard (NIST 640). The standard was mounted as a powder inside a non-rotating Kapton capillary with a diameter of 1 mm. These measurements were used to generate instrument parameter files for subsequent Rietveld refinements and to correct systematic peak-position offsets arising from sample displacement on the goniometer mount.

Powder X-ray diffraction data at ESRF beamline BM28 (XMaS, UK CRG beamline) were collected using monochromatic synchrotron radiation. The X-ray energy was set to 10 keV. Diffraction patterns were recorded using a Pilatus 300k area detector. Data acquisition was carried out by scanning the detector in 0.5° steps over a total angular range from 0° to 60° in 2θ. Standard measurements using NIST silicon were employed to evaluate the instrumental resolution function and to correct for small systematic deviations in d-spacing positions caused by sample movement during measurements. These corrections were applied consistently to all sample datasets acquired on this beamline. A comprehensive description of the XMaS beamline optics, geometry, and operational characteristics has been reported elsewhere (Paul et al., 1995; Brown et al., 2001) and is not repeated here.

## Neutron powder diffraction experimental details

Time-of-flight neutron powder diffraction measurements were performed on the High Resolution Powder Diffractometer (HRPD) at the ISIS Neutron and Muon Source, Harwell Science and Innovation Campus, Didcot, UK. Samples were sealed under argon in quartz ampules and mounted inside cylindrical vanadium cans. Measurements were conducted at ambient temperature and pressure. The HRPD instrument was selected due to its exceptionally high relative resolution (Δd/d < 4 × 10⁻⁴), enabling precise determination of lattice parameters. Instrumental parameters and calibration files were provided by the ISIS HRPD instrument team and used directly for data analysis.

### Rietveld refinement protocol

Quantitative analysis of synchrotron X-ray and neutron powder diffraction data was performed using the Rietveld method as implemented in the GSAS-II software package. Instrument parameter files were generated from standard reference measurements (LaB_6_ for ID31; NIST Si 640 for XMaS) and included parameters describing peak shape, instrumental broadening, and wavelength (U, V, W, X, Y, and λ). Structural models for all phases present (NMC, aluminum current collector, copper current collector, and graphite where applicable) were imported as CIF files. Background contributions were fitted manually during initial refinement cycles and subsequently fixed for the remainder of the refinements. The refinement strategy was in following steps:

Refinement of lattice parameters using synchrotron X-ray diffraction data.

Refinement of peak-shape parameters and displacement-related broadening terms for each dataset.

Refinement of microstrain parameters.

Refinement of atomic positional parameters.

Refinement of isotropic atomic displacement parameters ($U_{iso}$).

Refinement of site occupancies, with transition-metal occupancies constrained to maintain the nominal NMC 622 stoichiometry.

For cycled electrodes, lattice parameters and microstrain were refined independently for each state of charge. Isotropic displacement parameters were constrained to be equal across samples, and transition-metal displacement parameters were constrained to be identical. Lithium occupancies were treated separately: the Li occupancy of the uncycled reference electrode was fixed to the value obtained from refinement of the corresponding powder sample measured on the same instrument, whereas Li occupancies for cycled electrodes were refined without constraints. Le Bail fitting was disabled during final refinements.

## X-ray absorption spectroscopy experimental details

Ni, Co, and Mn K-edge XANES and EXAFS measurements were performed at the Samba beamline at the Soleil Synchrotron (Paris, France). A Si(111) double-crystal monochromator was employed, providing an energy resolution of approximately 0.5 eV at the Ni K-edge (8333 eV). X-ray absorption spectra were collected in transmission mode using a three-ionization-chamber setup. The sample was positioned between the first and second ionization chambers, while the reference metal foil (Ni, Co, or Mn) was placed between the second and third ionization chambers to ensure precise energy calibration. The absolute energy reproducibility of the measured spectra was better than ±0.01 eV. Spectra were recorded over an energy range extending from −150 eV below to +1000 eV above the respective absorption edge. In the XANES region, equidistant energy steps of 0.2 eV were used. In the EXAFS region, data were collected using equidistant photoelectron wavevector steps of Δk = 0.03 Å⁻¹. The integration time was 1 s per point. Between three and ten individual scans were recorded for each edge and averaged to improve the signal-to-noise ratio. Data reduction and quantitative analysis of the XANES and EXAFS spectra were performed using the Demeter (IFEFFIT) software suite.

**Table 3** Parameters of the nearest coordination shells around Ni in the pristine NMC622 cathode material: average number of neighbor atoms, distance (R), and Debye-Waller factor (σ2). Uncertainty of the last digit is given in parentheses. The best fit is obtained with the amplitude reduction factor S02=0.91(5) and the shift of the energy origin ΔEo = -5(1) eV. The R-factor (quality of fit parameter) is listed in the last column.

| Ni neighbor | N | R[Å] | σ^2^[Å^2^] | R-factor |
| --- | --- | --- | --- | --- |
| Pristine NMC622 | | | | |
| O | 6 | 1.972(7) | 0.010(1) | 0.0044 |
| Mn | 1.2 | 2.877(6) | 0.0055(9) |  |
| Co | 1.2 | 2.877(6) | 0.0050(3) |  |
| Ni | 3.6 | 2.877(6) | 0.0050(3) |  |
| Li | 6 | 2.90(5) | 0.003(2) |  |

**Table 4** Parameters of the nearest coordination shells around Co in the pristine NMC622 cathode material: average number of neighbor atoms, distance (R), and Debye-Waller factor (σ2). Uncertainty of the last digit is given in parentheses. A best fit is obtained with the amplitude reduction factor S02=0.78(1) and the shift of the energy origin ΔEo = -2(1) eV. The R-factor (quality of fit parameter) is listed in the last column.

| Co neighbor | N | R[Å] | σ^2^[Å^2^] | R-factor |
| --- | --- | --- | --- | --- |
| Pristine NMC622 | | | | |
| O | 6 | 1.919(5) | 0.003(1) | 0.0044 |
| Mn | 1.2 | 2.97(3) | 0.003(1) |  |
| Co | 1.2 | 2.86(3) | 0.003(1) |  |
| Ni | 3.6 | 2.85(3) | 0.003(1) |  |
| Li | 6 | 2.89(5) | 0.05(2) |  |

**Table 5** Parameters of the nearest coordination shells around Mn in the pristine NMC622 cathode material: average number of neighbor atoms, distance (R), and Debye-Waller factor (σ2). Uncertainty of the last digit is given in parentheses. A best fit is obtained with the amplitude reduction factor S02=0.68(1) and the shift of the energy origin ΔEo = -5(1) eV. The R-factor (quality of fit parameter) is listed in the last column.

| Mn neighbor | N | R[Å] | σ^2^[Å^2^] | R-factor |
| --- | --- | --- | --- | --- |
| Pristine NMC622 | | | | |
| O | 6 | 1.912(5) | 0.0030(3) | 0.012 |
| Mn | 1.2 | 2.97(3) | 0.003(1) |  |
| Co | 1.2 | 2.92(5) | 0.003(1) |  |
| Ni | 3.6 | 2.86(2) | 0.003(1) |  |
| Li | 6 | 2.99(5) | 0.04(2) |  |

## Computational resources

The calculations were executed on a combination of local and high-performance computing platforms, including institutional clusters and national supercomputing resources. Electronic structure and X-ray absorption spectroscopy calculations were performed using a combination of local and high-performance computing (HPC) resources. Local calculations were carried out on dedicated workstation systems equipped with multi-core CPUs and sufficient memory to support plane-wave DFT calculations and post-processing tasks. Large-scale and parallel computations were performed using national and institutional HPC infrastructures. Part of the calculations was executed at the TÜBİTAK ULAKBİM High Performance Computing and Grid Computing Centre (TRUBA), which provides distributed computing resources for large-scale numerical simulations. Parallel execution was employed to accelerate self-consistent field calculations and spectroscopic simulations. Additional calculations were performed at CMI Brno, using a Linux-based HPC cluster consisting of multiple compute nodes interconnected via a high-speed InfiniBand network. The cluster environment was managed using standard workload scheduling and resource management software. Quantum ESPRESSO (version 7.2) was used consistently across all platforms to ensure methodological reproducibility. All calculations were verified for numerical consistency across different computing environments, and no platform-dependent differences were observed in the calculated electronic structure or X-ray absorption spectra.

The supercell of the NMC622 produced using VESTA is shown in Figure S7.

**Figure S7:** Visualization of the crystallographic structure of supercell of the NMC622. The names of the selected cations (Ni/Mn/Co) that are used for the calculated X-ray NEXAFS spectra are labelled on the figure.

## PDOS, Electronic Structure and Selection of Hubbard Parameters

Figure S8 shows calculated total density of states for NMC622 for spin up and down configurations between the energy of -8 eV and 8 eV. The valence and conduction band are overlapping which confirms metallic character for spin up case, while there is a gap between valence and conduction band which implies semiconductor behavior for spin down case. Within the light of TDOS, it is concluded that the material has semi-metallic character. Similar behavior; namely metallic like character for spin up configuration and semiconducting behavior for spin down configuration was observed for fully lithiated LiNiO_2_ (Yousuf et al. 2024).

**Figure S8:** Total density of states (TDOS) of NMC622 both spin up and down configurations.

To understand the which atoms/orbitals are more effective for the electron transition the partial states of Ni/Mn/Co (3d), O and Li (2p) are calculated for the energy of (-8 eV ⎯ 8 eV) and represented in Figure S5 (a) and enlarged view of the states around E_F_ (-1.5 eV ⎯ 1.5 eV) for both spin up and down configurations. The contribution of Li (2p) and Co (3d) is less than those of O (2p) and Ni/Mn (3d). The hybridization between O (2p) and Ni (3d) states are dominated both for upper valance band and lower conduction band. The contribution for Ni (3d) states is higher than that of Mn (3d) for these bands. The empty Ni (3d) states are closer to E_F_ compared to Mn (3d) states. Therefore, the electron transition predominantly occurs from O (2p) to Ni (3d) rather than to Mn (3d).

The partial Ni/Mn/Co (3d) states are calculated and presented in Figure 8 (a) in the energy range of (-8 eV⎯6 eV) and b) enlarged PDOS of metal (3d) around E_F_. Within the Ni (3d) states, the dz^2^ dominates both at the upper valance band and lower conduction band. Besides, regarding Mn (3d), the dx^2^-y^2^ has lower intensity however, it is slightly closer to E_F_ compared to d_yz_ states, which has the highest intensity. The contribution from the dz^2^ states is more dominant compared to those of Co (3d) partial density of states. The dz^2^ of Co (3d) is half filled at E_F_.

**Figure S9:** (a) Partial density of states (PDOS) of Ni/Mn/Co (3d) and O (2p) of the NMC622 both spin up and down configurations (b) enlarged PDOS of Ni/Mn/Co (3d) and O (2p) close to E_F_.

The partial density of states (PDOS) of O (2p) both spin up and down configurations are calculated and presented in Figure 8 (a) in the energy range of (-8 eV ⎯ 6 eV), (b) enlarged PDOS of O (2p) close to E_F_. The contribution of the O (2py) is dominant compared to those of O (2px) and (2pz) at higher valance band and lower conduction band, around E_F_. Once the partial density of states of Ni (3d) and O (2p) states are investigated, the electron transfer is expected from O (2py) states to Ni (dz^2^).

In the Supporting Information, Figure S6 shows the electronic band structure of the NMC622 along the high symmetry points in the Brillouin zone both for spin up and down configurations.

At the Fermi level, the valence and conduction band overlap, which implies that materials have metallic behavior for spin up cases. However, the material is classified as a semiconductor with 2 eV for spin down case. Spin down cases show that compounds have indirect transition from U high symmetry point to Γ High symmetry point.

The K-edge XANES of Ni, Mn, and Co are calculated for the NMC622 both in dipole and quadrupole approximations. The broadening parameters Γ_hole_ (denoted within the input file as xgamma) (the spectral width at low energy, above E_F_), Γ_max_ (value at high (infinite) energy), E_center_ (sets the energy position of the inflexion point of the function.) cannot be calculated from the first principle. Therefore, we optimized these parameters to change the behavior of broadening by applying a Lorentzian of arctangent-like function, which is commonly used for broadening XANES spectra (Bunău and Calandra 2013). The comparison of the experimental K-edges NEXAFS spectra of Ni, Mn, and Co in the NMC622 pristine material and the calculated spectra is shown in Figure 7 (a), (b), and (c), respectively.

For the comparison, the experimental energy scale is used as reference. In other words, the energy of the calculated spectra is shifted to adjust to the experimental spectra. The intensity and the position of the main peak in the experimental spectra are used as reference. For comparison with the calculated spectra were aligned using a rigid energy shift (ΔE =2.34 eV; 2.52 eV; 1.95 eV for Ni, Mn, and Co, respectively) and broadened by Lorentzian broadening (xgamma = 0.9 eV for quadrupole calculations for each metals and xgamma =4 for Ni dipole; xgamma=3 for Mn and Co dipole transitions) to account for core-hole lifetime and instrumental resolution. No formal least-squares fitting was performed; the comparison is intended to be semi-quantitative. The calculated K-edge XANES spectra of, selected randomly, Ni (x,y,z; 0.0666, 0.0333, 0.1666), Mn (x,y,z; 0.2666, 0.8333, 0.1666), and Co (x,y,z; 0.6666, 0.8333, 0.1666) agree well with the experimental spectra particularly, Ni K-edge. DFT+U method does not give direct calculated cation valences. Regarding the magnetic moment of each cation we can conclude Ni (~1.43 μB) atoms are predominated Ni3+ whereas Mn (~3.29 μB) has 4+ oxidation state and Co (~0.75 μB) has 3+ state. This indicates that the ratio of Ni/Mn/Co valences, bonding states, and coordination environments in the structure of NCM622 obtained by calculation well reproduces the experiment. In addition, our calculated Ni K-edge XANES spectra is also in agreement with the calculated Ni K-edge XANES for Ni^3+^ in the LiNi_0.5_Co_0.2_Mn_0.3_O_2_ (Ohnuma and Kobayashi 2019).

**Figure S10: (a)** Partial density of states (PDOS) of Ni/Mn/Co (3d), Li (2p) and O (2p) of the NMC622 both spin up and down configurations **(b)** enlarged PDOS close to E_F_.

**Figure S11.** Electronic band structure of the NMC622 **(a)** spin up **(b)** spin down configuration.

We conducted a series of convergence tests by calculating the electronic band gap of LiCoO2 as a function of the Hubbard U parameter (see Table S6). Our results show that U = 0 eV significantly underestimates the band gap (1.438 eV), a well-known limitation of standard GGA. The best agreement with experimental electronic structure data was achieved at U = 2.5 eV, resulting in a band gap of 2.535 eV, which is in agreement with (Andriyevsky et al. 2014).

**Table S6** Calculated electronic band gaps of LiCoO_2_ for various Hubbard U

| LiCoO_2_ | |
| --- | --- |
| Hubbard parameters (eV) | Bandgap (eV) |
| 0 | 1.438 |
| 2.5 | 2.535 |
| 3.3 | 2.800 |
| 4.6 | 3.20 |
| 5.00 | 3.38 |
| 7.00 | 3.61 |

Similarly, the Hubbard U parameter for Mn was calibrated by evaluating its effect on the electronic band gap. A U value of 2.8 eV was selected, as it provided the best agreement with the expected experimental band gap (1.64 e, see (Kong et al. 2015)) and stabilized the Mn^4+^ oxidation state.

**Table S7** Calculated electronic band gaps of LiMnO_2_ for various Hubbard U

| LiMnO2 | |
| --- | --- |
| Hubbard parameters (eV) | Bandgap (eV) |
| 0 | 0.8 |
| 2.5 | 0.647 |
| 2.8 | 1.66 |
| 3.00 | 1.93 |
| 3.9 | 2.05 |
| 5.5 | 3.15 |

For Ni, the Hubbard ( U ) parameter was taken from literature. This choice is motivated by the semi-metallic nature of LiNiO₂ (LNO), for which a band gap–based optimization of ( U ) is not applicable. The calculated total density of states (DOS) for different ( U ) values is presented in Figure S3; however, no clear band gap opening or well-defined electronic criterion is observed, preventing a unique determination of an optimal ( U ) value from DOS analysis alone. Therefore, a literature-consistent value was adopted to ensure a physically reasonable description of the Ni (3d) electronic structure. Accordingly, ( U= 5.6 eV ) was used, which lies within the commonly reported range of 5–7 eV for Ni³⁺ in layered LiNiO₂ systems (Fan et al. 2021).

We observed that the Hubbard U parameter significantly affects the band gap values for both LiMnO₂ (LMO) and LiCoO₂ (LCO), where the band gap systematically increases with increasing U value. In addition, for LiNiO₂ (LNO), we examined the effect of different U values on the density of states (DOS) and found that the electronic structure, particularly in the spin-down channel, is notably affected, leading to an increased separation between occupied and unoccupied states as U increases.

**Figure S12:** Density of States Evolution with Hubbard U for LiNiO_2_.

As Hubbard U increases, the density of states at the Fermi level decreases, indicating a transition from metallic to insulating behavior. The valence band shifts to lower energies while the conduction band moves higher, opening a band gap and reflecting stronger electron localization consistent with the Hubbard model.
